# Emerging Roles of the Unique Molecular Chaperone Cosmc in the Regulation of Health and Disease

**DOI:** 10.3390/biom12121732

**Published:** 2022-11-23

**Authors:** Ting Xiang, Muchuan Qiao, Jiangbo Xie, Zheng Li, Hailong Xie

**Affiliations:** 1 Hunan Province Key Laboratory of Tumor cellular Molecular Pathology, Cancer Research Institute, Heng yang School of Medicine, University of South China, Hengyang 421009, China; 2 The Affiliated Cancer Hospital of Xiangya School of Medicine, Central South University, Hunan Cancer Hospital, Changsha 410013, China; 3 Laboratory for Functional Glycomics, College of Life Sciences, Northwest University, Xi’an 710069, China

**Keywords:** Cosmc, C1GALT1, chaperonin, O-glycosylation, Tn antigen

## Abstract

The core-1 β1-3galactosyltransferase-specific chaperone 1 (Cosmc) is a unique molecular chaperone of core-1 β1-3galactosyltransferase(C1GALT1), which typically functions inside the endoplasmic reticulum (ER). Cosmc helps C1GALT1 to fold correctly and maintain activity. It also participates in the synthesis of the T antigen, O-glycan, together with C1GALT1. Cosmc is a multifaceted molecule with a wide range of roles and functions. It involves platelet production and the regulation of immune cell function. Besides that, the loss of function of Cosmc also facilitates the development of several diseases, such as inflammation diseases, immune-mediated diseases, and cancer. It suggests that Cosmc is a critical control point in diseases and that it should be regarded as a potential target for oncotherapy. It is essential to fully comprehend Cosmc’s roles, as they may provide critical information about its involvement in disease development and pathogenesis. In this review, we summarize the recent progress in understanding the role of Cosmc in normal development and diseases.

## 1. Introduction

**Figure 1:**
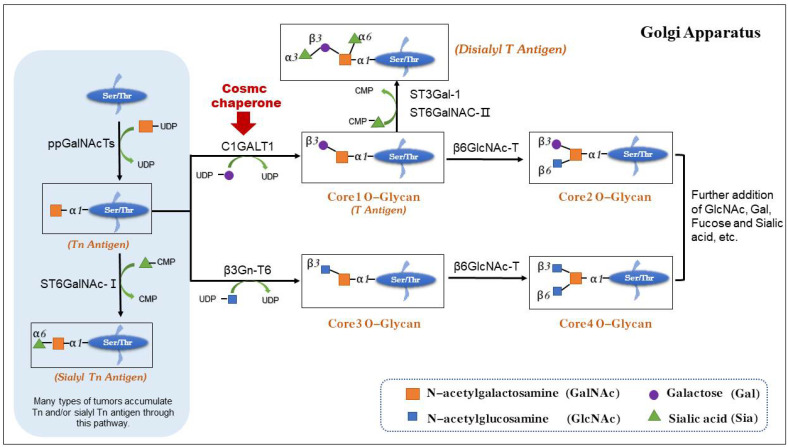
Schematic diagram of the biosynthesis of O-glycan. O-glycosylation is normally initiated in the Golgi apparatus. With the assistance of the molecular chaperone Cosmc, C1GALT1 adds Gal from UDP-Gal to the Tn antigen to form the T antigen, which is extended by adding other sugars to produce normal O-glycans. Without Cosmc, the function of C1GALT1 would be lost, resulting in the generation of Tn and/or Sialyl Tn antigen, which is usually associated with the molecular mechanisms of the development of various diseases or tumors.

Glycosylation is the most common post-translational modification of proteins in eukaryotic cells [1]. O-glycosylation (serine/threonine-linked) and N-glycosylation (Asn-linked) are two common forms of protein glycosylation in mammals [2]. O-glycosylation is a protein modification that occurs in secreted and membrane-bound proteins and plays a very important role in many life activities, such as ontogeny [3] and cellular communication [4]. Abnormal O-glycosylation modification is usually associated with the development of many diseases [5] and tumors [6]. O-GalNAc glycosylation is a type of O-glycosylation, and the most structurally complex type of protein glycosylation modification [7]. More than 80% of cell membrane proteins and extracellularly secreted proteins are O-GalNAc-glycosylated proteins [8]. O-GalNAc glycosylation is activated by the transfer of the alpha conformation of GalNAc on UDP-GalNAc to serine or threonine residues in the presence of α-N-acetylgalactosaminyltransferase, resulting in the formation of α-O-GalNAc-Ser/Thr (Tn antigen), a common precursor of mucin-type O-glycans [6]. There are eight commonly O-GalNAc-modified core structures [7,9], with the most abundant being the Core-1 O-glycan Gal1-3GalNAc1-Ser/Thr (T antigen) [10]. The T antigen is synthesized with the cooperation of C1GALT1 and its unique molecular chaperone, Cosmc [11]. Under the action of C1GALT1 and Cosmc, galactose is added to Tn antigen to generate T antigen, which can be further modified or extended to form the complex O-glycans by downstream glycosyltransferases in the Golgi [12,13,14] (Figure 1).

While the sequence of amino acids determines the stability of the protein, correct folding is essential for protein conformation and function, which requires the assistance of a molecular chaperone [15]. Cosmc plays a crucial role in many biological functions, including mediating platelet production [16], affecting the function of immune cells [17], and kidney development [3]. In addition, Tn antigen and STn antigen are products of aberrant glycosylated expression, and their aberrant expression is partly due to the impaired expression of C1GALT1 and/or Cosmc [18]. The impaired function of Cosmc has been found in a variety of human diseases, including human cancers [19]. Cancer angiogenesis [20] and epithelial–mesenchymal transition (EMT) [21] may all be aided by abnormal Cosmc expression on the surface of tumor cells. Regulation of O-glycosylation can be achieved by regulating the enzymes and molecular chaperones involved in O-glycan synthesis. As the key to O-glycosylation, Cosmc may be used as a therapeutic target for tumors and various diseases. Interestingly, our understanding of Cosmc has grown steadily in recent years, owing to its potential for developing new diagnostic and therapeutic procedures for common and serious chaperonopathies, such as various types of cancer and autoimmune diseases. This review focuses on current knowledge of the Cosmc in normal development and various diseases.

## 2. The Structure and Function of Cosmc

**Figure 2:**
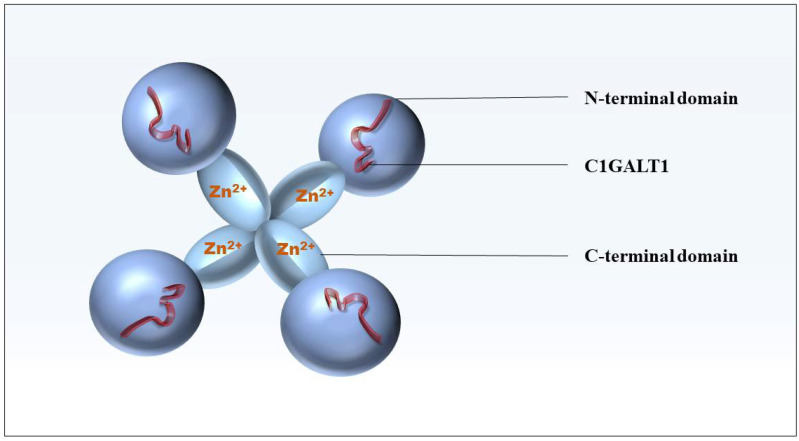
Model of Cosmc structure. The N-terminal domain of Cosmc (dark blue) is responsible for interacting with C1GALT1 (red peptide) for chaperone function, and the C-terminal domain (light blue) mediates oligomerization and Zn^2+^ binding.

The Cosmc gene, also known as C1GALT1C1, is an important molecular chaperone for the formation of active C1GALT1 [22]. The specificity of Cosmc for C1GALT1 probably stems from their unique co-evolutionary history. There is 26% homology in the amino acid sequence between human Cosmc and human C1GALT1 [23,24]. The Cosmc consists of two relatively independent domains, the N-terminal domain and the C-terminal domain. The N-terminal domain of Cosmc mediates chaperone function in vitro and binds directly to C1GALT1, while the C-terminal domain mediates oligomerization and Zn^2+^ binding and may play a regulatory role in vivo (Figure 2) [25]. In ER, Zn^2+^ regulates calreticulin and cadherin activities, and Zn^2+^ binding can induce co-partner binding [26]. Therefore, the high affinity of Cosmc for Zn^2+^ could increase its activity as a C1GALT1 chaperone protein. In addition, Cosmc is a type II unidirectional transmembrane protein localized to the ER. The determinant of the localization of Cosmc to the ER is related to the transmembrane domain (TMD), which consists of 18 amino acids and also enables Cosmc to acquire the ability of ER retention. In contrast, the mutation of a single Cys residue in the TMD of Cosmc prevents the formation of disulfide bond dimers in Cosmc and eliminates ER retention [27]. This also provides new insights into the molecular mechanism by which TMDS resident in ER proteins promotes ER localization.

**Figure 3:**
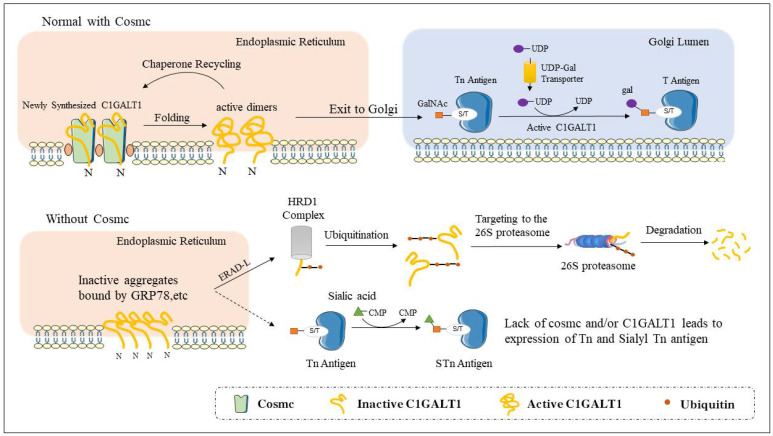
Working model for Cosmc function. Human Cosmc is located in the ER, where it interacts with the activated C1GALT1. Activated C1GALT1 is inserted into the Golgi apparatus and is involved in the synthesis of core-1 O-glycans (T antigens). When Cosmc is mutated and dysfunctional, misfolded C1GALT1 aggregates in the endoplasmic reticulum and is retrotranslocated from the endoplasmic reticulum to the cytosol, where it is ubiquitinated and degraded by the proteasomal machinery.

Although the Cosmc protein itself does not possess galactosyltransferase activity, the formation of an effective and stable C1GALT1 must be accompanied by the presence of Cosmc [28]. During the folding of C1GALT1, Cosmc in the ER recognizes the newly synthesized C1GALT1 polypeptide chain [29] to prevent C1GALT1 misfolding [23], resulting in the formation of active C1GALT1. At the same time, during this process, the luminal domain of Cosmc can also interact with the refolded C1GALT1 either directly in the free or covalent form but not in the active dimeric form, resulting in the formation of a relatively stable complex of Cosmc and refolded C1GALT1 while forming the reactivated C1GALT1 in an ATP-independent manner, forming a binding/release cycle [30,31]. Finally, the active C1GALT1 is transported to the Golgi apparatus to participate in O-glycosylation [30] (Figure 3).

Cosmc is an ATP-bound resident ER protein, while C1GALT1 is a Golgi resident protein. Other than Cosmc, it has been found that there is no other partner in the ER that can fold C1GALT1, but glucose-regulating protein 78 (GRP78) can produce co-immunoprecipitation with inactive C1GALT1 produced in the absence of Cosmc [32]. In the ER, GRP78 can interact with unfolded proteins to help them exit [33]. Thus, without Cosmc, unfolded C1GALT1 is retained in the ER lumen and interacts with GRP78 to form a misfolded complex, which then reverse-translocates back to the cytoplasm and is ubiquitylated and degraded by the proteasomal machinery in the 26S proteasome [14,34]. Since the lesion in C1GALT1 is located in the luminal domain, this misfolded C1GALT1 is likely to enter the endoplasmic reticulum-associated degradation luminal (ERAD-L) pathway for degradation [35]. The HRD1 complex is shown to be the mechanism for the reverse-translocation of ERAD-L misfolded proteins, which may involve the reverse transcription of inactive C1GALT1 from the ER to the cytoplasm, followed by ubiquitination target destruction [36] (Figure 3). However, other mechanisms underlying the degradation of type II transmembrane proteins in the ER remain to be explored.

At present, the structural and chaperone functions of Cosmc are still in the preliminary stage. Further studies are necessary to reveal more structural functions of Cosmc and the interaction between Cosmc and C1GALT1.

## 3. Roles of Cosmc in Normal Development

Glycosylation is one of the most complex and diverse post-translational modifications. This large glycan diversity leads to a wide range of biological functions. Cosmc, which plays an important role in O-glycosylation, has also been studied and shown to play multiple regulatory roles in normal human growth and development (Table 1).

### 3.1. Cosmc Affects Platelet Production

Cosmc and the extension of O-glycan play an important role in the expression and function of platelet adhesion proteins [16]. The efficiency of Cosmc is lethal to mice embryos, and mouse develop thrombocytopenia and macrothrombocytosis. Compared to normal mice, Cosmc-deficient platelets have impaired platelet GPIbα expression and function, which may be related to the Cosmc-induced loss of galactose on core-1 O-glycan that leads to the loss of platelet GPIbα core 2 O-glycan [16]. While GPIbα is the major platelet von Willebrand factor (VWF) receptor [40], the absence of Cosmc affects the functional binding of platelets to VWF. In addition, GPIbα has a high-affinity binding site for α-thrombin and accounts for most of the total α-thrombin that can bind with platelets, leading to platelet adhesion and spreading, secretion, and aggregation [38]. Therefore, defective GPIbα expression also affects thrombin signaling [41]. Platelets from mice after the knockout of Cosmc showed impaired activation of integrin αIIbβ3, suggesting that the inactivation of GPIbα caused by the deletion of Cosmc also leads to defects in the thrombin-induced activation of key platelet glycoproteins [16], and the widespread expression of the Tn antigen caused by Cosmc deficiency. Furthermore, the presence of symptoms such as thrombocytopenia and bleeding are consistent with the symptomatic manifestations of Tn syndrome, which suggests that Cosmc is closely related to the pathogenesis of Tn syndrome [22].

### 3.2. Cosmc Affects Kidney Development

The disruption of mucin-type O-linked glycosylation has been shown to impair renal function [42]. Podocytes are highly differentiated epithelial cells of the glomerular basement membrane, involved in maintaining the structure and function of the glomerular filtration barrier and playing an active role in preventing plasma proteins from entering the urinary ultrafiltrate [43]. Cosmc, an essential molecular chaperone in C1GalT1-mediated mucin-type O-linked glycosylation, has been shown to be essential for podocytes [3]. Mice with Cosmc knocked out of their podocytes show signs of proteinuria, glomerulosclerosis, and renal failure, which are thought to be associated with the loss of podocyte-associated proteins and the loss of podocytes [3]. The absence of Cosmc causes a downregulation of the expression levels of the podocalyxin and podoplanin proteins, which are closely related to the integrity of the glomerular filtration barrier. Podocalyxin is an essential protein for normal podocyte development in mice and humans, and it is the major Tn antigen-containing protein in podocytes lacking Cosmc [3]. Furthermore, the loss of podocytes in mice knocked out of Cosmc can be compensated for by neighboring podocytes expressing Cosmc, similar to the cellular nonautonomous mechanism that has been proposed to maintain podocyte structural integrity and for which Cosmc-mediated mucin-type O-glycoprotein is important for maintenance [3]. These results suggest that some pathological changes in the kidney may be associated with a decrease in Cosmc activity, which is independent of intrinsic defects and the influence of immune factors.

### 3.3. Cosmc Affects the Function of Immune Cells

#### 3.3.1. Cosmc and Extended O-glycosylation Are Key Factors Controlling B-Cell Homing and Maintaining B-Cell Immune Tolerance

B cells have a variety of immune functions, and due to their ability to produce antibodies, B cells are mainly considered active regulators of immune responses and are major contributors to the pathogenesis of immune-related diseases [44,45]. Cosmc-mediated O-glycosylation may play a key role in B-cell development and homing [17]. It has been shown that smc-deficient mice show dynamic changes in the frequency and absolute number of B-spectrum progenitors, suggesting that Cosmc is required for the normal progressive development of B cells in the bone marrow [17]. The C-C motif chemokine ligand 21 (CCL21) is significantly reduced in Cosmc-deficient B cells compared to normal B cells [17]. CCL21 is a ligand for C-C chemokine receptor 7 (CCR7), which is a key molecule in B-cell homing, and it has a potential extracellular N-terminal O-glycosylation site. The loss of extended O-glycan may affect its functional impact [46]. When multiple chemokine receptors are desensitized, lymphocytes exhibit impaired blockage and subsequent reduced homing [47]. Therefore, it is speculated that impaired chemokine responsiveness caused by the lack of Cosmc may be related to the lack of sialylated extended O-glycans. Although Cosmc deficiency is known to lead to markedly impaired chemokine signaling and thus impaired B-cell homing, the O-glycan molecules on B cells and their potential recognition partners within the endothelium have yet to be explored in more detail.

In addition, the B-cell receptor (BCR) on the surface of B cells is responsible for recognizing and binding antigens and transmitting antigen-stimulating signals, and pathologically increased BCR signals contribute to B-cell hyperactivity and autoimmunity [48,49]. The Cosmc-deficient mice spontaneously exhibit pathologies such as autoimmune disease, which may result from the fact that Cosmc deficiency prolongs BCR retention on the cell surface and promotes stronger BCR signaling, thus causing B cells to overreact to stimuli [37]. This suggests that the absence of core-1 O-glycan on B cells prolongs the surface retention of BCR, which contributes to enhanced BCR signaling [37]. Therefore, it can be hypothesized that Cosmc and its homologous core-1 O-glycan on B cells could act as an important immune checkpoint for maintaining B cell tolerance to prevent the development of pathogenic auto-reactive B cells.

#### 3.3.2. Cosmc and Extended O-glycosylation Are Key Factors in Maintaining Peripheral T Cells

T lymphocytes are derived from bone marrow progenitor cells, and the generation of functional T cell receptors in the thymus through genomic rearrangements is an important process in T cell maturation. This is followed by the settlement of T cells in peripheral lymphoid organs with blood circulation, leading to an immune response to antigenic stimuli [50]. It has been shown that T cells require extended O-glycosylation for proper physiological functioning in the thymus and SLO [38]. The absence of T cell Cosmc not only leads to a significant reduction in the number of peripheral T cells in the spleen and lymph nodes but also to a disproportionate loss of T cells expressing Tn antigen on their surface, suggesting that Cosmc is critical for T cell persistence in the blood or SLO [38]. Interestingly, although the deletion of Cosmc reduced the number of T cells, it did not interfere with the maturation process of T cells. Cotransfer experiments revealed that T cell-specific Cosmc knock out cells have reduced T cell ability to home to SLO and are not maintained in circulation, which may be mediated by the improper glycosylation due to a dysfunction in the adhesion molecule, L-selectin (CD62L) [38]. These results demonstrate that Cosmc, as well as extended O-glycosylation, are key factors in establishing and maintaining peripheral T cell populations.

#### 3.3.3. Cosmc and Extended O-glycosylation Mediate Phagocytosis of Apoptotic Cells by Macrophages

The macrophages, originating from progenitor cells in the bone marrow, are essential for the effective control and clearance of infections and for promoting tissue repair and wound healing [51]. In programmed cell death, cells undergoing apoptosis are phagocytosed by macrophages to avoid the release of harmful substances [52]. T cell immunoglobulin and mucin domain-containing molecule 4 (Tim4) binds apoptotic cells mainly through its immunoglobulin structural domain, recognizing phosphatidylserine, which mediates the phagocytosis of apoptotic cells by macrophages [52]. The mucin-like region of Tim4 has been reported to contain several highly O-glycosylated sites, and aberrant O-glycosylation affects the stable expression of Tim4 and, consequently, the clearance of apoptotic cells [39]. It has been shown that resident peritoneal macrophages (rpMacs) from Cosmc knock out mice exhibit impaired phagocytosis of apoptotic cells, but macrophage differentiation and numbers are not affected. The aberrant glycosylation caused by the deletion of Cosmc reduces the protein expression level of Tim4, which is related to its possible attack by protein hydrolysis [39]. Otherwise, Cosmc deletion does not cause the abnormal expression of other cytosolic-related genes in macrophages, suggesting that Cosmc-mediated core-1 O-glycan is required for Tim4-dependent normal cytosolic action and may contribute to stable expression of Tim4 glycoprotein [39].

## 4. Roles of Cosmc in Non-neoplastic Diseases

Cosmc has been shown to play an important role in normal development. Here, we discuss the common diseases associated with Cosmc and the roles that it plays in various diseases (Table 2).

### 4.1. Immune Diseases

#### 4.1.1. Immunoglobulin A Nephropathy (IgAN)

IgAN is the most common primary glomerulonephritis worldwide and usually presents with a progressive decline in renal function, resulting in high morbidity and mortality [59]. The prevalence of IgAN is highest in developed Asian countries, where it is 40–50% [60]. There is increasing evidence that abnormally glycosylated immunoglobulin A1 (IgA1) molecules, primarily IgA1 lacking galactose in the circulation, are the trigger for thylakoid deposition and subsequent renal injury in IgAN [61]. The IgA1 O-glycosylation site is located in the hinge region, and the O-glycan is a core-1 structure, usually linked to GalNAc and galactose [62]. The addition of galactose is mediated by C1GAlT1. The stability of the C1GAlT1 protein depends on its interaction with the molecular chaperone Cosmc. The absence of Cosmc leads to the rapid degradation of the C1GAlT1 protein. Galactose cannot be attached to GalNAc, resulting in abnormal O-glycosylation [22].

Qin [54] et al. found that the expression level of Cosmc in B lymphocytes of IgAN patients was significantly downregulated, which was related to the abnormal O-glycosylation level in IgAN. The downregulation of Cosmc expression leads to galactose deficiency by affecting the stability of the C1GAlT1 protein. Galactose-deficient O-glycan forms antigen–antibody complexes with IgG antibodies against the hinge region of IgA1 [63,64] and forms pathogenic circulating immune complexes that can evade the clearance of desialoglycoproteins by liver receptors [65,66]. The complexes are eventually deposited in the glomerular thylakoid region, leading to glomerular damage [67].

Some studies have shown that, when IgAN patients have severe renal insufficiency, the T-adjuvant 2 cytokine interleukin-4 (IL-4) is oversecreted, leading to the downregulation of Cosmc mRNA expression [68]. The downregulation of Cosmc expression in the lymphocytes of IgAN patients may be related to the hypermethylation of the Cosmc gene promoter induced by IL-4 [53].

#### 4.1.2. Tn Syndrome

In addition to IgAN, another disorder strongly associated with reduced Cosmc activity is Tn syndrome. Tn syndrome is characterized by the expression of Tn antigens in the blood cell subsets of patients [69]. Patients with Tn syndrome may present asymptomatically or with clinical symptoms such as hemolytic anemia, thrombocytopenia, and occasional bleeding, which are usually considered to be caused by Tn antigens recognized by anti-Tn antibodies and applied to leukocytes or platelets [70]. In addition, glycoproteins on platelets or leukocytes are extremely important for cell function, and changes in glycoprotein glycosylation status may affect their function [12].

Wang et al. [16] found that endothelial cells and hematopoietic cells in Cosmc-KO mice showed bleeding and thrombocytopenia while obviously lacking C1GALT1 activity. Decreased C1GALT1 activity results in Tn antigen expression on mouse platelets. This suggests that thrombocytopenia and bleeding in Tn syndrome patients are related to Cosmc-mediated impaired platelet function. In recent years, genetic evidence has shown that the major genetic basis of Tn syndrome is caused by acquired somatic mutations of Cosmc in blood progenitor cells, which can lead to the misalignment of the open reading frame and even the premature termination of transcription, leading to severe or complete impairment of their chaperone function [22]. Mi et al. [18] found that hypermethylation of the Cosmc promoter leads to the inactivation of C1GALT1 and the expression of Tn and/or STn antigens [19]. Cosmc provides an alternative mechanism for the abnormal expression of Tn antigen, which may have important implications for understanding the abnormal expression of the Tn antigen in Tn syndrome.

Fully understanding the molecular mechanism of Cosmc in Tn syndrome should aid in the development of new diagnostic techniques and therapeutic approaches.

### 4.2. Inflammatory Diseases

#### 4.2.1. Lung Inflammation

It has been reported that endothelial cells and hematopoietic cells lacking C1GALT1 exhibit extreme rolling and recruitment disorders in inflammatory tissues, which are associated with reduced E-selectin-mediated neutrophil adhesion. This suggests that aberrant O-glycosylation affects the critical early stages of inflammation [71]. Cytokine signaling and inflammatory responses, on the other hand, can influence mucin O-glycosylation by activating intracellular signaling pathways [72,73].

Inflammatory lung disease can thicken airway mucus not only because of the evaporation of airway water but also because of the increased glycosylation of airway mucin 5AC [74]. As the main secretion of mucin is by the airway epithelium, the glycosylated branch chain of airway mucin 5AC is modified by O-glycosylation [75]. T antigen is the most common precursor of mucin-type O-glycan, which can be found on membrane-bound and secreted glycoproteins [32], and its synthesis depends on the expression of C1GALT1 and Cosmc [23]. Previous studies have shown that the expression of T antigen and Cosmc is increased in the airway epithelial cells of patients with chronic inflammatory pneumonia, suggesting that Cosmc may be involved in airway mucin glycosylation in patients with pneumonia by regulating T antigen [55]. Neutrophil elastase (NE), an important inflammatory factor secreted by neutrophils, is known as the most potent mucus agonist [76]. Lin et al. [55] found that NE increased the expression of T antigen by promoting the expression of Cosmc and C1GALT1 activity, while NE did not increase the expression of T antigen in the absence of Cosmc expression. In addition, NE stimulation activates PI3K via the EGFR/RAS pathway, and PI3K activation increases Cosmc expression, C1GALT1 activity, and T antigen expression [55]. These findings suggest that Cosmc and PI3K play an important role in the signaling pathway of T antigen overexpression induced by NE.

In conclusion, Cosmc not only directly regulates T antigens involved in inflammatory lung disease, but it also serves as an important regulatory point for inflammatory factors that regulate airway mucin O-glycosylation. Therefore, targeting Cosmc to reduce the over-modified O-glycosylation level of airway mucin 5AC and altering the rheology of airway mucus provides a new idea for the development of drugs to improve the symptoms of airway mucus obstruction in patients with airway inflammatory diseases.

#### 4.2.2. Bowel Inflammation

The strongest association between chronic inflammation and malignant disease is the development of colon cancer in individuals with inflammatory bowel disease (IBD) [77]. IBD is a chronic inflammatory disease that includes ulcerative colitis and Crohn’s disease [78], and the pathogenic mechanism is related to abnormal immune activation of intestinal bacteria and intestinal microbiota dysbiosis [79].

**Figure 4:**
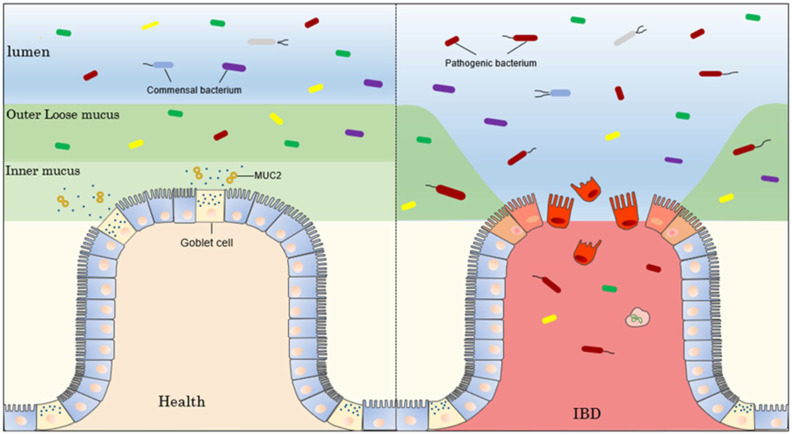
The colon has a two-layered mucus system; the outer layer is infiltrated by intestinal bacteria, and the inner layer is unaffected by bacteria and physically separates them from the epithelium. The lack of defects in this inner MUC2 mucin, which is the main mucus component of the intestine, disrupts the protective properties of the inner colonic mucus layer, allowing direct contact between bacteria and epithelial cells to cause inflammation and bleeding.

The mucosal surface of the colon is protected by a natural immune barrier, the mucus barrier, between the mucosal surface and the intestinal lumen [80]. The colonic mucus layer is divided into sterile inner mucus and outer loose mucus occupied by bacteria [81]. MUC2 mucin secreted by goblet cells is the main component of the colonic mucus layer [82] (Figure 4). O-glycans account for 80% of the amount of MUC2 mucin, which usually depends on Cosmc [83]. Mucin glycosylation is required for mucin expression and function, and O-glycan deficiency has been linked to spontaneous colitis in mice [84]. This suggests that the loss of Cosmc function may also be a molecular mechanism in the pathogenesis of IBD.

Genetic background is thought to be involved in the pathophysiology of IBD. Genome-wide association studies identified Cosmc on the X chromosome as a risk factor for IBD [85]. It has been shown that the deletion of the Cosmc allele in male mice leads to damage to the mucus layer and the induction of spontaneous, microbial-dependent inflammation. Unexpectedly, female mice were protected from inflammation. This suggests that Cosmc is a sex-specific risk gene for IBD [56].

It has been shown that intestinal Cosmc deficiency leads to a significant reduction in gut microbiota diversity [56], which may lead to increased inflammatory and immune responses [86,87]. The reason why Cosmc affects the differential distribution of intestinal microbiota is related to the differential regulation of gene expression in different intestinal regions by Cosmc through an indirect mechanism downstream of glycoprotein [56]. In addition, Cosmc directly regulates glycosyl synthesis and promotes the expression of bacterial host ligands, selecting only symbionts in the colon. This suggests that Cosmc functions as a region-specific spatial regulator of gut microbiota, which is essential for maintaining intestinal stability [56]. In conclusion, Cosmc may be a breakthrough point for further studies of IBD and may help to reveal more pathogenic mechanisms related to IBD.

### 4.3. Neurodegenerative Diseases

Alzheimer’s disease (AD) is a progressive neurodegenerative disease that impairs cognitive function and is a major cause of dementia [88]. AD is divided into early-onset AD and late-onset AD (LOAD) [89]. The pathogenesis of AD are complex and diverse, among which abnormal O-glycosylation is involved in the pathogenesis of AD and affects the progression of AD [90].

Tn antigen expression is increased in the cortex of AD patients [91], a finding supported by Frenkel [92]. Cosmc mutations leading to Tn antigen expression have been shown to cause a variety of diseases [12]. Recent studies have shown that Cosmc has a p.D131E mutation in the coding region of LOAD subjects [57], which leads to a significant decrease in C1GALT1 activity and a significant increase in C1GALT1 protein expression in LOAD subjects. This may be caused by the failure of Cosmc to clear the aggregated, misfolded C1GALT1 protein [57]. This suggests that the Cosmc mutation initiates abnormal galactosylation. It is worth noting that, similar to the coding region, single nucleotide polymorphisms and promoter region modifications also cause changes in Cosmc expression [57]. Therefore, the correlation between Cosmc promoter mutations and AD warrants future investigations.

### 4.4. Viral Diseases

A key step in the viral life cycle is transmission to new target cells, thereby initiating infection [93], and viral synapse formation is the cell-to-cell transmission mechanism used by retroviruses [94]. Human T cell leukemia virus type 1 (HTLV-1) hijacks cellular proteins to establish cell–cell contact zones, called virological synapses (VS), and infects target cells during replication [94]. It has been shown that the expression levels of the sialic acid proteins CD43 and phosphatase CD45 in lymphocytes play a key role in HTLV-1 infection, and these antigens are highly O-glycosylated in normal cells. After knocking down Cosmc, the density and negative charge of CD43 and CD45 on T lymphocytes decreased, and the level of HTLV-1 infection decreased significantly [58]. This suggests that Cosmc plays an important role in the cell-to-cell transmission of the virus. Therefore, it is promising to develop effective drugs against Cosmc to prevent the spread of viral infections.

## 5. Cosmc Function in Tumorigenesis

### 5.1. Regulation of Cell Proliferation

During tumor progression, cancer cells acquire many characteristic alterations, including the unregulated proliferation of tumor cells [95]. Normal cell growth and maintenance require an appropriate balance of O-GlcNAcylation. An abnormal level of O-glycosylation is closely associated with tumor growth and differentiation [12].

It has been shown that the deletion of function caused by the hypermethylation of the Cosmc promoter increases the expression levels of the proliferative genes Ki67 and proliferating cell nuclear antigen (PCNA) in breast cancer (BC) cells [96]. The high levels of Ki67 expression in BC are thought to be associated with estrogen-negative, Her2-positive, and axillary lymph node involvement in BC [97]. This is not only limited to BC, as the proliferation marker Ki-67 is also localized throughout the organotypic epithelium in Cosmc hypermethylated pancreatic cancer cells, whereas unmutated pancreatic cancer cells showed Ki-67 only in basal cells, suggesting that loss of function due to Cosmc hypermethylation could induce pancreatic cancer cell proliferation [98].

The AKT/mTOR signaling pathway has been reported to regulate not only tumor cell migration [99], but also tumor cell proliferation [99]. The loss of O-GalNAc glycan resulting from the loss of Cosmc function has been shown to significantly affect the AKT/mTOR pathway in pancreatic ductal adenocarcinoma (PDAC) and significantly enhance the growth of PDAC cells [99]. Moreover, abnormal glycosylation caused by the loss of Cosmc function can also lead to the expression and/or loss of function of MUC2, the main intestinal secreted mucin, thereby inducing oncogenic properties of colon cancer (CRC), such as the upregulation of tumor cell proliferation [100]. However, in ovarian cancer (OVCA), Cosmc deletion not only promotes the apoptosis of OVCA cells but also leads to the decreased proliferation of OVCA cells [101], which is consistent with the research result that the downregulation of C1GALT1 leads to decreased growth of OVCA cells [102].

As an important player in O-glycosylation modification, Cosmc has been shown to regulate the O-glycosylation of vascular endothelial growth factor receptor-2 (VEGFR2) to increase VEGFR2 activity in hemangiomas [103]. VEGFR2 is a receptor that mediates the endothelial cell response to VEGF, and the upregulation of VEGFR2-dependent signaling in hemangioma endothelial cells has been demonstrated [104]. Rare VEGFR2 mutations aside, VEGFR2 activity is thought to be a key determinant of abnormal hemangioma cell growth [103]. Moreover, the Cosmc regulation of VEGFR2 O-glycosylation also enhanced the phosphorylation of AKT and ERK, two major signaling pathways known to regulate HUVEC proliferation in human umbilical vein endothelial cells [103]. This suggests that Cosmc can be involved in the mechanism of hemangioma development by increasing endothelial cell proliferation.

From the above, it is clear that Cosmc plays different roles in various tumors. Therefore, it is necessary to study its mechanism in-depth in various tumors.

### 5.2. Regulation of Cell Apoptosis

Dysregulated apoptosis is associated with a variety of diseases, including human cancer. The tumor necrosis factor-related apoptosis-inducing ligand (TRAIL) can be produced and secreted by most normal tissue cells, and its receptors, death receptor 4 (DR4) and death receptor 5 (DR5), are membrane glycoproteins containing O-glycosylation sites. Upon their binding to TRAIL, the cytoplasmic domain of the death receptor interacts with the junction molecule Fas-associated death domain to transmit apoptotic signals, thereby triggering apoptosis [105].

Apoptosis is associated with altered glycosylation patterns [106]. It has been shown that DR4/DR5 cells carrying sialic acid–T antigens are more sensitive to TRAIL than cells carrying Tn/STn antigens, and the main mechanism responsible for this differential sensitivity is the promotion of homo-oligomerization of death receptors through O-glycan structures on death receptors DR4/DR5 or other glycoproteins on the cell surface [107]. Cosmc can promote the homo-oligomerization of DR4/DR5 via the extended sialic acid–T antigen, which is essential for death signaling, and oligomers of DR5 can also induce apoptosis independently of the presence of TRAIL [107]. Cells with dysfunctional Cosmc express truncated O-glycan and Tn/STn antigens, and in this case, Tn/STn antigens on DR4/DR5 glycoproteins not only prevent the homo-oligomerization of DR4/DR5, but also promote hetero-oligomerization between DR5 and decoy receptors2 lacking the death domain, thereby attenuating the death signal of DR5 [107]. This idea was also confirmed by Ding et al [108]. Cosmc transfection significantly suppressed the malignant behavior of colon cancer cells and enhanced TRAIL-induced apoptosis by correcting aberrant O-glycosylation [108]. In addition, the deletion of Cosmc function resulting in Tn and STn antigens also promotes cancer progression in BC [96] and pancreatic cancer [109], which includes the reduced apoptosis of tumor cells, suggesting that Cosmc can affect cancer progression by regulating Tn and STn antigens.

Apoptosis plays an important role in cancer therapy and is a major effector function of many anti-cancer therapies. The role of Cosmc in apoptosis suggests it as a new target for cancer therapy.

### 5.3. Regulation of Cell Migration

Metastasis is a major cause of cancer-related death. Epithelial–mesenchymal transition (EMT) is a developmental program that has been conserved throughout evolution [110]. Aberrant activation of EMT gives tumor cells enhanced metastatic potential, and it also leads to the acquisition of therapeutic resistance in tumor cells, posing a major clinical challenge to cancer therapy [111].

The basic characteristics of EMT can be specified by the expression of specific epithelial and mesenchymal marker proteins. The negative expression of E-cadherin and the strong positive expression of N-cadherin, the so-called “cadherin-switch”, have been reported in tumor metastasis [112]. This “cadherin-switch” was also observed in the tumor tissues of mice implanted with Cosmc-deficient PDAC cells, suggesting that Cosmc enhances the invasiveness of PDAC cells by inducing EMT [21]. In colon cancer, the Cosmc deletion of the Tn antigen has been shown to activate the EMT pathway, and the upregulation of H-RAS may be the driver of EMT activation by the Tn antigen [113]. H-RAS is a member of the Ras guanosine triphosphatase family, and ectopic expression of oncogenic H-RAS has been shown to activate EMT, leading to increased invasiveness [114,115]. These results suggest that Cosmc may be involved in the metastasis mechanism of tumor cells by regulating various signaling pathways.

Cancer stem cells (CSCs) are the main cause of cancer aggressiveness, drug resistance, and tumor recurrence [116]. EMT has been shown to induce a CSC-like phenotype. Cells with the EMT phenotype influence the molecular characteristics of CSCs, and CSCs also express the EMT phenotype [117]. It has been reported that the glycosylation state of CD133 plays a key role in maintaining stem cell characteristics [118], and sialylation regulates the stability of CD133 in cancer cells [119]. Meanwhile, CD44 is a CSC surface marker and one of the main carriers of truncated O-glycans [120]. Abnormal glycosylation caused by Cosmc deletion can cause EMT in PDAC cells and enhance the expression levels of stem cell markers CD44 and CD133 on PDAC cells [21], which may be one of the mechanisms by which cosmc regulates the stemness of tumor cells and mediates cancer metastasis. In addition, cancer cell stemness not only synergizes with EMT to promote cancer metastasis but is also closely associated with tumor drug resistance [117]. Given the effect of Cosmc on cell stemness, it would be interesting to further explore the relationships between Cosmc and tumor drug resistance [117].

### 5.4. Regulation of Immune Surveillance

The body’s immune system increases the adaptability of tumor cells to immune escape while killing them due to the activation of various immunosuppressive pathways by the tumor cells themselves [121]. The glycosylation of cell surface glycoproteins and glycolipids is one of the main features of tumor cells. It has been suggested that the specific glycan profile on tumor cells can be considered a novel immune checkpoint [122]. Tumor cells have a different “glycosylation coating” compared to normal cells, and their glycosylation response can affect the function of antigen-presenting cells (APCs) and alter T cell differentiation and natural killer (NK) cells’ activity to lead to immune escape [122]. C-type lectins can bind to sugars and are mainly found on APCs, where galactose-type lectins (MGL) are selectively expressed by immature dendritic cells (DCs) and macrophages, which can bind to GalNAc terminal residues and the Tn antigen [123,124,125]. The triggering of Tn-specific MGL receptors on APC has been shown to drive immunosuppression through the induction of apoptosis and the production of tolerogenic cytokines [126,127]. It has been shown that the deletion of Cosmc actively promotes Tn antigen expression. Previous research has also shown that the Tn antigen can be activated by interacting with DC cells in the APC and MGL2 on macrophages, stimulating DC cells to induce the secretion of large amounts of IL-10 and tumor necrosis factor-α, and directing T cell differentiation to a regulatory T cell phenotypes in an IL-10 or tumor necrosis factor-dependent manner, thereby activating the immune evasion mechanism of lung tumor cells [128].

Aberrant glycosylation may alter charge distribution, conformational dynamics, and the volume of space occupied by mucins, and it is expected to have a significant impact on cellular interactions, such as contacts with effector cells (NK cells and cytotoxic T lymphocytes) [129]. It has been shown that mucopolysaccharide extension beyond Tn antigens alters their sensitivity to NK cell- and cytotoxic T lymphocyte-mediated killing [130]. This was demonstrated in breast cancer (T47D) cells and pancreatic cancer cells (Capan-1), and the knockdown of Cosmc in the cells inhibited polysaccharide extension over Tn antigen, thereby increasing the susceptibility of both tumor cells to NK cell-mediated antibody-dependent cellular cytotoxicity (ADCC) and cytotoxic T lymphocyte-mediated killing [130]. The study speculates that this is because both ADCC and cytotoxic T lymphocytes require immune synapse formation to function and that highly glycosylated mucins interfere with synapse formation between tumor cells and NK cells [131]. The truncated O-glycan resulting from the deletion of Cosmc affects the surface expression level, function, and/or interaction of proteins in the synapse, leading to enhanced cell killing sensitivity.

### 5.5. Regulation of Angiogenesis

Cancer cells require new angiogenesis for tumor expansion and metastatic spread [132]. Cosmc knockdown in lung cancer cells induces high tumor vascularization due to the VEGF secreted by Tn antigen-expressing tumors, which promotes vascular endothelial cell migration and tubulogenesis in vitro [128]. Induced angiogenesis involves the recognition of MGL2 cells, and the recognition of tumor-expressed Tn antigens by MGL2 cells induces APCs to produce VEGF or other pro-angiogenic factors [128]. Tumor-associated macrophages have been reported to produce pro-angiogenic factors, matrix metalloproteinases, and vascular constructs to promote angiogenesis, thereby ensuring the provision of oxygen and nutrients to solid tumor cells [133]. In addition, hypoxia, or inflammation-induced alterations in endothelial cell glycosylation may also favor angiogenesis and metastasis [134]. Future studies should examine whether Cosmc directly regulates the neovascular system that is active during tumorigenesis in vivo.

### 5.6. Cosmc as Novel Prognosis Biomarker

In light of this, Cosmc can regulate the development of a variety of cancers, and is associated with many cancer hallmarks, such as cell proliferation, invasion, and metastasis. Therefore, revealing the effective potential diagnosis and prognostic value of Cosmc as a tumor biomarker is important for clinical decision-making.

Recently, by performing a clinical analysis of the correlation between Cosmc expression and the prognosis of hepatocellular carcinoma (HCC), Shen [135] found that Cosmc expression was upregulated in paired or unpaired non-tumor tissues compared to hepatocellular carcinoma tissues. Furthermore, Cosmc expression is strongly negatively correlated with vascular invasion and tumor differentiation, suggesting that Cosmc has antitumor properties in HCC. Not only that, but survival analysis also confirms that a low expression of the Cosmc is associated with shorter survival. Subsequently, Shen developed a new clinical prediction model for hepatocellular carcinoma based on risk factors identified in multiple regression, such as Cosmc expression, vascular invasion, and TNM stage, and validated it using an external cohort from the TCGA database, which showed that Cosmc expression can improve the ability to predict HCC prognosis [135].

Experiments demonstrate the role of Cosmc as a clinical biomarker and therapeutic target for tumors. However, more experimental studies are still needed to determine the reliability and validity of Cosmc in combination with other conventional markers for cancer diagnosis and prognosis.

## 6. Final Remarks

The extensive investigation of Cosmc has yielded complex molecular insights into its regulation and function in normal physiological and disease states. As an important molecular chaperone in O-glycosylation, Cosmc plays an important role in assisting the synthesis of C1GALT1. The dysregulation or dysfunction of Cosmc is associated with immune diseases, infections, neurodegenerative diseases, and cancer. The significance of Cosmc in a wide range of pathophysiological conditions not only makes this protein a promising target for the treatment of a variety of diseases, but also makes this protein a potential cancer diagnostic and prognostic biomarker. In addition to the diagnostic biomarkers of IgAN, Cosmc is also expected to be a potential prognostic biomarker for HCC. In the course of Cosmc studies, evaluation of Cosmc through animal models, patient samples, and experimental systems has expanded our understanding of this protein and its role in health and disease. However, most of the current studies focus on the mechanistic verification of Cosmc. Therefore, it is necessary to combine basic laboratory research with clinical studies in order to translate experimental results into clinical application and practice. Further studies of Cosmc in the future will reveal the mechanism that unerlies Cosmc’s multiple functions.

## Figures and Tables

**Table 1:** Roles of Cosmc in normal development.

Location	Funtion	Citation
Platelet	Mediates platelet production by affecting the expression and function of platelet glycoproteins.	[16]
Kidney	Maintains the normal function of podocyte cells in the kidney.	[3]
B cell	Mediate the homing of B cells by affecting chemokines and can maintain the immune tolerance of B cells.	[17,37]
T cell	Mediates the homing of T cells and maintains the presence of peripheral T cells.	[38]
Macrophage cell	Affect the phagocytosis of apoptotic cells by macrophages.	[39]

**Table 2:** Mechanism of Cosmc in non-neoplastic diseases.

Diseases	Effects	Citation
Immunoglobulin A Nephropathy	Downregulation of Cosmc expression causes abnormal glycosylation of IgA1, which is involved in the pathogenesis of IGAN.	[53,54]
Tn syndrome	Loss of Cosmc function causes abnormal expression of Tn antigens, resulting in Tn syndrome.	[16,18]
Inflammatory pneumonia	Cosmc regulates the glycosylation of airway mucin 5AC via T antigen and plays an important role in the stimulation of T antigen overexpression by the inflammatory factor neutrophil elastase.	[55]
Inflammatory bowel disease	Cosmc spatially regulates the intestinal microbiota in a region-specific manner, and its functional deficiency causes a decrease in intestinal mucosal MUC2 protein, causing IBD with a sex-specific profile.	[56]
Alzheimer’s disease	Cosmc mutations cause abnormal glycosylation in late-onset AD and affect the progression of AD.	[57]
HTLV-1 infection	Cosmc enhances HTLV-1 virus infection between cells by affecting the glycosylation of CD43 and CD45.	[58]

## Data Availability

Not applicable.
